# Enhanced Camera Relocalization Through Optimized Accelerated Coordinate Encoding Network and Pose Solver

**DOI:** 10.3390/s25061920

**Published:** 2025-03-19

**Authors:** Xinbo Chai, Zhen Yang, Xinrong Tan, Mengyang Zhu, Changbin Zhong, Jianping Shi

**Affiliations:** 1 School of Electrical and Automation Engineering, Nanjing Normal University, Nanjing 210046, China; 231812078@njnu.edu.cn (X.C.); 241812081@njnu.edu.cn (X.T.); 231812035@njnu.edu.cn (M.Z.); 231812043@njnu.edu.cn (C.Z.); 2 School of Instrument and Engineering, Southeast University, Nanjing 210096, China; 223367@seu.edu.cn

**Keywords:** ACE network, scene-aware, camera relocation, pose calculation

## Abstract

This paper presents an improved approach for scene-aware camera relocalization using RGB images and poses. Building upon the ACE network, we proposed a refined head structure that integrates skip and dense connections alongside channel attention mechanisms. Additionally, we introduced modifications to the loss function and pose solver, leveraging SQPnP and iterative optimization. These enhancements led to significant improvements in the localization accuracy and speed, as evidenced by our experiments on the 7scenes, 12scenes, and wayspots datasets. Here, we show that the average localization errors were reduced by up to 30% and the computational times were cut by approximately 10% compared to the original ACE network, demonstrating the practicality and robustness of our approach.

## 1. Introduction

Visual relocalization is a task within the field of computer vision; it is aimed at determining the camera’s position and orientation (i.e., its location and direction) by analyzing visual information within a scene. Currently, the use of camera relocalization algorithms to predict six-degrees-of-freedom camera poses from input images plays an important role in various application areas, such as autonomous driving, robotics, and augmented reality (AR)/mixed reality (MR). Camera relocalization methods can be mainly divided into two broad categories: those based on regression and those based on structure. Early relocalization techniques primarily relied on regression-based methods, which could directly deduce camera poses from images; however, due to their susceptibility to image retrieval influences, they offered a lower accuracy, leading to a trend towards structure-based camera relocalization methods [1]. Structure-based camera relocalization methods can primarily be divided into two stages: establishing a coordinate mapping relationship between 2D pixel coordinates and 3D spatial coordinates through matching or regression, and then estimating the camera pose using the perspective-n-point (PnP) [2] series of pose-solving methods in conjunction with the random sample consensus (RANSAC) [3] algorithm.

Structure-based camera relocalization methods consist of two types: sparse feature matching and scene coordinate regression. Sparse feature matching relies on using local descriptors to establish correspondences between 2D map inputs and a given explicit 3D model, mainly comprising two steps: feature detection with feature detectors and feature decoding with descriptors. The learning approaches can be classified into three types based on the sequence of these steps: detect-then-describe, detect-and-describe-simultaneously, and describe-then-detect [4]. Scene coordinate regression eliminates the need for explicit 3D map construction and descriptor extraction, enabling an implicit transformation to be learned from 2D pixels to 3D point coordinates, which is relatively more efficient. Although scene coordinate regression can achieve an accuracy and relocalization times comparable to sparse feature matching, this method requires retraining with data when applied to new scenes, making it imperative to accelerate the retraining process as much as possible [5]. To address this, Brachmann et al. proposed the accelerated coordinate encoding (ACE) network architecture [6], which divides the network into a scene-agnostic backbone and a scene-specific head. When introducing new images, only the head needs to be retrained, effectively achieving the goal of accelerated coordinate encoding.

On one hand, to maintain the advantages of accelerated coordinate encoding, this paper retained the use of the pretrained, scene-transferable backbone from the original model and the concept of decorrelating image feature gradients. On the other hand, to achieve a more efficient and accurate camera coordinate computation, we improved the network structure of the model’s head, the computational scheme of the loss component, and the calculation method and early exit conditions for the pose solver.

Firstly, we designed a new network structure for the head section that predicts pixel 3D coordinates with a higher precision and robustness. Subsequently, we modified the loss computation scheme to reduce the computational complexity. Next, we introduced a new pose-solving scheme that, in conjunction with the original approach, enables the pose solver to achieve an equivalent accuracy at a faster rate. Lastly, our experiments indicated that, compared to the previous ACE network, our improved network can achieve camera relocalization with a greater precision and at a faster pace in the majority of scenarios.

## 2. Related Work

### 2.1. Feature Extraction

Feature extraction is a key step in the fields of computer vision and image processing, serving to extract useful feature information from raw data to support subsequent tasks such as classification, detection, and recognition. Initially, feature extraction relied on manually designed methods such as edge detection and corner detection, which possess invariance and distinctiveness, requiring extensive expert knowledge and a targeted design [7]. Subsequently, with the development of deep learning, data-driven feature learning has become mainstream. Early classic models such as LeNet [8], AlexNet [9], and VGG [10] that utilize convolutional neural networks learn feature representations from raw data, overcoming the limitations of manual feature extraction and making feature detection learning and usage more efficient and automated. In recent years, with the increase in computing resources and the prevalence of large-scale datasets, transfer learning using pre-trained models has become increasingly popular. For instance, models such as ResNet [11] and EfficientNet [12] include a wealth of pre-trained weights available online, and utilizing these pre-trained weights can significantly enhance the performance for specific tasks while reducing data requirements and computational costs. The backbone used in our model, derived from the ACE model, consisted of eleven convolutional layers and two skip connections. The convolutional layer weights were obtained from a week-long pretraining on the first 100 scenes of the ScanNet dataset [13], enabling the rapid and effective extraction of crucial feature information from images. Moreover, for scene localization, the information from the image feature extraction part is transferable across scenes, and not affected by scene variations in terms of the localization accuracy. Therefore, this structure can effectively reduce the training time required for new scenes, better meeting the practical demands of application scenarios.

### 2.2. Coordinate Regression

Coordinate regression refers to the generation of 3D coordinates from input images, with conventional methods including random forest regression algorithms and deep learning neural networks. The random forest regression algorithm is a tree-based regression method that constructs multiple uncorrelated decision trees by randomly sampling data and features, obtaining prediction results in parallel. Each decision tree yields a predictive outcome based on the sampled data and features; by aggregating and averaging the results of all trees, the overall regression prediction of the forest is obtained. Deep learning neural networks establish an end-to-end coordinate mapping relationship between 2D pixel coordinates and 3D spatial coordinates and can be divided into two types as mentioned above: sparse feature matching and scene coordinate regression methods [14]. Scene coordinate regression methods, such as SANet [15]; DSAC [16] and its derivatives, DSAC++ [17] and DSAC* [18]; KFNet [19]; etc., are deep learning neural network approaches that implicitly establish coordinate mapping relationships. Compared to sparse feature matching methods that explicitly create 3D models, these approaches not only serve the purpose of privacy protection, but they also have lower storage requirements [20]. The main drawback of scene coordinate regression methods is the considerable time required for mapping in new scenes [21]. The ACE network architecture significantly reduces the training time for new scenes, effectively addressing this issue. Our proposed improvements enabled the model to achieve faster localization speeds during testing while maintaining a training duration similar to that of the ACE, making it better suited to the demands of practical scene localization.

### 2.3. Pose Estimation

Pose estimation relies on the fundamental principles of analytical geometry, deducing a camera’s external parameters through known 3D points and their corresponding points in the camera image [22]. The PnP solution method can estimate the camera pose from the 3D spatial coordinates of at least three known points on an object and their corresponding 2D-pixel coordinates. With the enhancement of computational capabilities, optimization algorithms have been increasingly incorporated into camera pose-estimation processes, such as estimating a camera pose by minimizing the reprojection error, thereby further improving the accuracy and robustness of camera pose estimation. Common PnP algorithms include ITERATIVE [23], [24], which refines the pose using the nonlinear Levenberg–Marquardt minimization scheme; squared quadratically constrained quadratic program for perspective-n-point (SQPnP) [25], which can quickly and globally optimize for the perspective-n-point problem; and accurate and practical three-point perspective (AP3P) [26], which is tailored for the three-point relocalization issue. Building upon this, the RANSAC algorithm was introduced, repeatedly sampling and fitting models to obtain the optimal model parameters, thus mitigating the adverse effects of noise and outliers, estimating the best inlier set, and endowing camera pose estimation with a greater precision and robustness. The pose solver we used originated from DSAC*, where the pose solver in DSAC* first randomly selects 64 sets of pixel points to relocalize the camera using the perspective-3-point (P3P) [27] method, and then scores the relocalization results, applying RANSAC’s pose optimization method to the highest-scoring result to involve as many valid predicted points as possible. We effectively improved this pose solver by altering the PnP pose-estimation approach and the iterative optimization process, ultimately achieving a faster execution of pose estimation.

## 3. Methods

### 3.1. Overview

**Figure 1:**
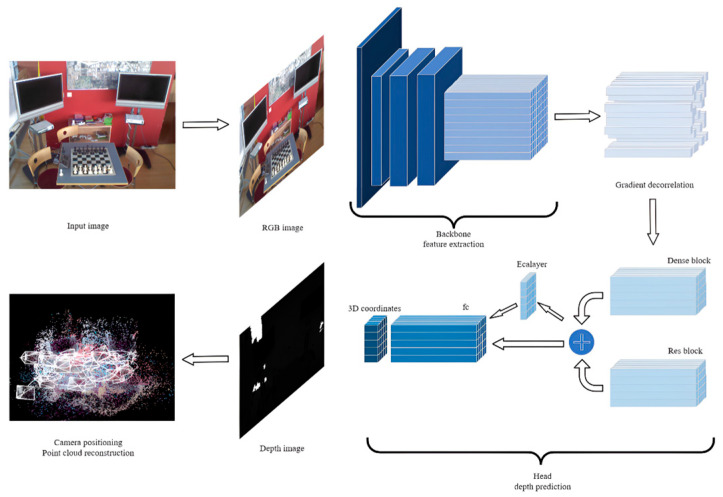
The model’s image processing workflow.

In Figure 1, we present the general processing flow of the model on images. Initially, the backbone part extracts and compresses feature information from the image, and then randomly selects some features to feed into the head, predicting the three-dimensional coordinates of pixels in the image. Finally, these coordinates are used to relocalize the camera that captured the image. If needed, the obtained three-dimensional coordinates can be used for scene reconstruction, which, although more time-consuming, allows for a more intuitive acquisition of scene information and the display of the camera pose.

Our improved model adopted the backbone part of the ACE network, which was trained for one week on the first 100 scenes of the ScanNet dataset, yielding a transferable feature extraction module. Given its superior performance and training costs, we chose not to attempt further modifications here. The enhancements made to the ACE network are described in the following subsections. Firstly, in Section 3.2, we detail the improvements to the head section of the ACE network, including the stacking of network layers, the types of modules referenced, the activation functions used, and the introduction of channel attention mechanisms. Then, in Section 3.3, we introduce improvements to the ACE loss function. On one hand, by modifying the computation sequence and logic, we ultimately reduced the computational complexity; on the other hand, by combining the Manhattan and Euclidean distances, we adapted the method to different datasets. Lastly, in Section 3.4, we present improvements to the pose solver. We not only changed the computation method for relocalizing the camera using predicted three-dimensional points, but we also added conditions for early termination during camera relocalization optimization, leading to both enhanced relocalization results and significantly shortened localization times.

### 3.2. Head Network Architecture

In the original ACE, the primary structure comprises eight 1 × 1 convolutional layers with an input and output channel count of 512 each, where skip connections are introduced after the third and sixth layers. Skip connections facilitate faster information propagation and gradient capture within the network, helping to address the problems of vanishing and exploding gradients. However, the sole use of serial 1 × 1 convolutions with skip connections still has its limitations, leading us to introduce dense connections in our network [28]. Dense connections not only reinforce gradient flow to mitigate the issue of gradient vanishing, but they also reduce the number of parameters, improving the parameter efficiency.

Initially, we replaced the three layers spanned by skip connections with three layers of dense connections, yet the test results indicated no improvement in the network performance, as the dense connections discarded some features preserved by skip connections. Therefore, considering the need to combine the strengths of both dense and skip connections, we proposed a parallel architecture incorporating three layers of both, allowing for the full exploitation of the advantages of each connection type to enhance the overall feature representation capabilities and optimization efficiency, enabling the model to perform well under various conditions.

Subsequently, after the dense and skip connections, we introduced a channel attention mechanism [29] by drawing on the ECA layer architecture using adaptive average pooling to weight the output channels, thereby further enhancing the feature representation ability and enabling the neural network to more effectively express key features in the data while reducing the impact of other noise.

Next, we switched all the activation functions in the network structure from ReLU to parametric ReLU (PReLU) [30]. The ReLU activation function used in the original model had its limitations, causing neuron death during training, whereas replacing it with PReLU can alleviate this issue, introducing a slight gradient in the negative region to maintain gradient flow.

Finally, the head network returns a 4D tensor $\left(\dot{x},\;\dot{y},\;\dot{z},\;\hat{w}\right)$, where $\dot{x},\;\dot{y},\;\dot{z}$ represent the homogeneous coordinates of the predicted three-dimensional scene, and the fourth element $\hat{w}$ is a non-normalized homogeneous parameter. To obtain the normalized homogeneous parameter w, preprocessing was applied to $\hat{w}$ using a biased and clipped Softplus operator, as defined by the following equation.

(1) $$w=\min\left(\frac{1}{S_{min}},\beta^{-1}\cdot \log\left(1+\exp\left(\beta \cdot \hat{w}\right)\right)+\frac{1}{S_{max}}\right),\;\beta =\frac{\log\left(2\right)}{1-S_{max}^{-1}}$$

**Figure 2:**
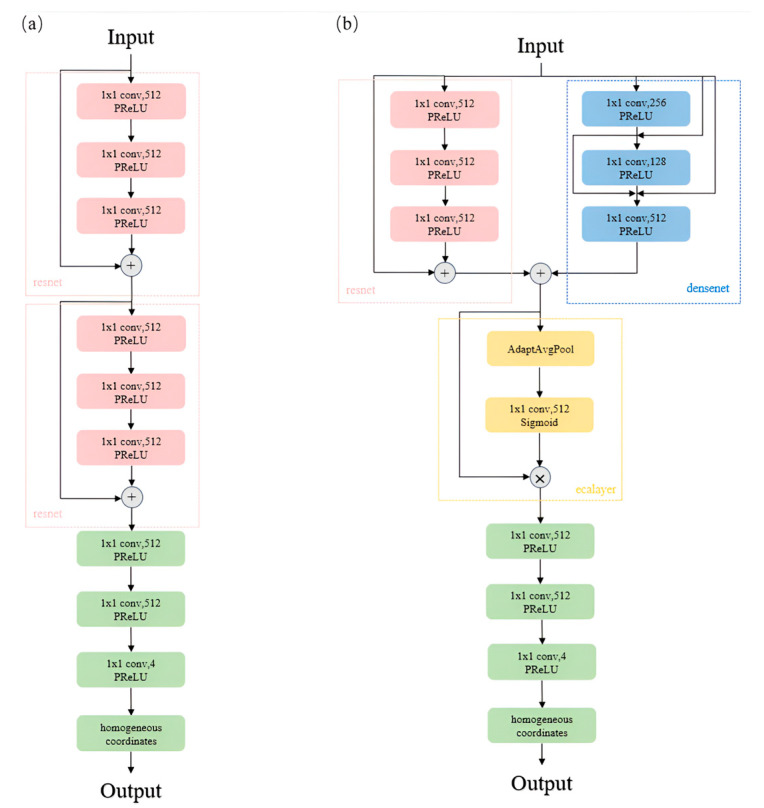
The head section of the original model (**a**). The modified head section (**b**).

Here, $S_{min}$ and $S_{max}$ are used to clip the value of the scaling factor determined by w, and w ensures that, when $\hat{w}$ is 0, the output homogeneous parameter w is 1. The normalized homogeneous parameter w, computed as described, enables the dehomogenization of the network’s output coordinates according to the formula $y=\frac{\dot{y}}{w}$, resulting in the predicted actual coordinates of the 3D scene. Figure 2 illustrates the head network structure of the meta-model and the improved results of the head network described in this section. It is evident that our designed network, while maintaining a similar parameter count, enhances the connectivity between its upper and lower layers, thereby more effectively extracting and transmitting image features.

### 3.3. Improvements to the Loss Function

In the original ACE framework, the loss function first converts predicted 3D coordinates into 2D pixel coordinates and computes the reprojection error, using $e_{\pi }$ to quantify the robust reprojection error for valid coordinate predictions. All points are classified into two categories based on hyperparameters, including the maximum depth threshold, minimum depth threshold, and maximum reprojection error threshold, resulting in two distinct loss computations. Points within the threshold apply a scaled tanh function to tighten the reprojection error distribution.

(2) $$\hat{e_{\pi }}\left(x_{i},\;y_{i},\;h_{i}^{*}\right)=\tau \left(t\right)\tanh\left(\frac{e_{\pi }\left(x_{i},\;y_{i},\;h_{i}^{*}\right)}{\tau \left(t\right)}\right)$$

The scaling factor for tanh is dynamically adjusted with a training-dependent threshold.

(3) $$\tau \left(t\right)=k\left(t\right)\tau_{\max}+\tau_{\min},\;\mathrm{with}\;\;k\left(t\right)=\sqrt{1-t^{2}}$$

In this approach, where  t∈(0,1) represents the relative training progress, the threshold is dynamically adjusted to gradually decrease as training progresses, enabling more effective model convergence and generalization. For points outside the threshold, the Manhattan distance between the predicted and actual three-dimensional coordinates is used as the loss. The two types of losses are summed and divided by the batch size to obtain the final loss value participating in the iteration.

Regarding this loss function, we revised the computation order to filter out some pixel points using the maximum and minimum depth thresholds first, thereby reducing the computational load of subsequent two-dimensional remapping errors. On the other hand, considering the occurrence of overfitting, we introduced an additional penalty term that incorporates the Euclidean distance for pixels outside the threshold in the loss function. The Manhattan distance measures the distance between two points in space along the coordinate axes, while the Euclidean distance calculates the straight-line distance between two points. The former tends to capture local variations, whereas the latter captures global variations. These two metrics are complementary, and combining them in the loss function enables the capture of multi-scale characteristics of the data. As a result, this improvement enhances the compatibility of the loss function across different datasets, leading to a better generalization performance.

### 3.4. Enhancements to the Pose Solver

In the original ACE, a pose solver from DSAC* is employed, which randomly selects 64 sets of four pixels with reprojection errors not exceeding the threshold for camera relocalization. The scoring of each relocalization result is performed according to Equation (4).

(4) $$\left(1-\frac{1}{1+e^{\frac{-5*\left(err-\tau_{in}\right)}{\tau_{in}}}}\right)*\frac{\alpha_{in}}{width\;*\;height}$$

In this approach, err denotes the current reprojection error value, while $\tau_{in}$ serves as the threshold for distinguishing between valid inliers and outliers. $\alpha_{in}$ quantifies the weighting factor of the inliers’ contributions to scoring, where the width and height represent the image height and width, respectively. The relocalization result with the highest score is selected for iterative pose refinement. Points are iteratively incorporated with reprojection errors within the threshold to refine the camera pose estimation. These refined pose estimates subsequently enhance the 3D point reprojection accuracy. Through successive refinements, the algorithm maximizes the number of inlier points until saturation is achieved.

Initially, we considered replacing the current pose solver with one from SRC [31]. However, experiments demonstrated that this solver’s performance was significantly inferior to that of the DSAC*-based solver. A code analysis revealed that, on one hand, this pose solver lacked a threshold constraint for the upper limit of depth error, leading to substantial shifts due to noisy data with large errors. On the other hand, its optimization logic involved calling all points with reprojection errors within the threshold for camera relocalization, iterating until the displacement between the current and previous reprojection results fell below the threshold. In contrast, the DSAC* pose solver ensured that the number of points used for camera relocalization exceeded that of the last iteration, allowing for more stable optimization.

Consequently, we decided to directly optimize the existing DSAC*-based pose solver. For four-point relocalization, we substituted the traditional P3P pose-solving method with the AP3P method, which introduced additional geometric constraints for more accurate, faster, and robust pose estimation. Subsequently, in terms of multi-point relocalization iterative optimization, we initially referred to SQPnP, using the SQPnP pose-estimation method as an optimization scheme for camera relocalization with multiple points within the reprojection error range, replacing the preceding nonlinear Levenberg–Marquardt minimization approach. Compared to the previous method, SQPnP offers a higher computational efficiency and is suitable for sparse feature point sets, significantly improving the model efficiency. Yet, our experiments indicated that directly utilizing SQPnP still had limitations, causing significant declines in localization precision and insufficient robustness in certain scenarios. Therefore, we further refined our approach by integrating the SQPnP pose-estimation method with the nonlinear Levenberg–Marquardt minimization scheme. Specifically, we employed the SQPnP algorithm when the iteration of the camera pose was rapid to achieve a quicker correction, while the nonlinear Levenberg–Marquardt scheme was used for slower iterations to correct the camera pose more accurately and robustly. Lastly, regarding the relocalization iteration conditions, we believe that an optimization criterion solely focusing on involving more points in reconstruction is not prudent. The inclusion of new points may cause significant shifts in the majority of the already-established points, implying erroneous shifts in camera pose estimation as well. Therefore, we recorded the average reprojection error of the points within the threshold for the current reprojection results. If the next iteration led to an average reprojection error exceeding a certain multiple of the previous error, it suggested that subsequent iterations would degrade the accuracy of the camera pose estimation, necessitating the early termination of the iteration process.

## 4. Experiments

**Figure 3:**
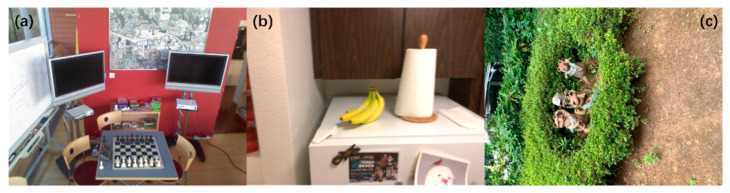
Example plots of the 7scenes (**a**), 12scenes (**b**), and wayspots (**c**) datasets.

In this section, we provide a detailed description of the network training process. First, in Section 4.1, we introduce the training details and network configuration. In Section 4.2, we present the performance of our improved model compared to the baseline ACE model on two indoor datasets, 7scenes [32] and 12scenes [33], as well as the outdoor dataset wayspots. The results demonstrate the enhancements of our model, with example images from the three datasets shown in Figure 3. Finally, in Section 4.3, we conducted isolated ablation studies on the two improvements, proving their necessity.

### 4.1. Experimental Setup

In the implementation of this work, for the feature extraction component, we retained the ACE backbone network and its pre-trained weights, with a batch size of 5120. For the training phase, we utilized the AdamW optimizer with learning rates ranging between $\left(2.5\times 10^{-4}\right)$ and ($7.5\times 10^{-3}$). In the pose-estimation component, we employed an improved DSAC* pose solver with 64 RANSAC hypotheses. Throughout the implementation process, data storage was maintained in a half-precision format as much as possible to reduce memory usage.

### 4.2. Quantitative Evaluation

Firstly, we compared the test results of various models previously proposed with those of our model on the 7scenes dataset. The 7scenes dataset comprises seven types of indoor localization scenes, providing 7000 mapped images for each scene with areas ranging from 1 m^3^ to 18 m^3^, making it a commonly used dataset for camera relocalization. Table 1 presents the discrepancies between the predicted and actual camera poses in the 7scenes dataset using different models. It is evident that, compared to previously proposed models, our model achieved a higher precision in camera relocalization tasks.

#### 4.2.1. Indoor Relocalization

Subsequently, we conducted a more detailed assessment of the improved model on the 7scenes dataset. Table 2 presents the assessment results comparing the improved and original models. The test results include the percentage of scenes with coordinate prediction errors within 10 cm/5 deg, 5 cm/5 deg, 2 cm/2 deg, and 1 cm/1 deg; the median of the camera coordinate prediction error in terms of the angle and distance; and the average time spent on the prediction. The results show that, in most scenarios, our model achieved a higher localization accuracy and a faster localization speed compared to the original model. Additionally, the median errors in the angle and distance predictions were mostly reduced. However, it is important to note that both the improved model and the original model performed poorly in certain scenarios, such as the “pumpkin” scene. This was likely due to interference from factors such as unnatural lighting in the dataset itself. Therefore, the optimization effectiveness should be comprehensively evaluated across multiple scenarios.

Subsequently, we replicated the experiments on the 12scenes dataset using the same metrics, with the assessment results of both models presented in Table 3 and Table 4. The results demonstrate that, compared to the original model, our model achieved a higher precision and a faster speed in relocalization across most indoor scenes, and it generalized well to different scenarios across various datasets. Furthermore, we observed that the same model achieved a higher relocalization accuracy on the 12scenes dataset compared to the 7scenes dataset, which may be attributed to the characteristics of the dataset itself. Under the premise of easily achieving high-precision localization, our improved model showed a relatively significant enhancement in relocalization speed. This reflects the effectiveness of our approach, which utilized SQPnP and the early termination of iterative pose solvers to reduce the localization time, particularly in scenarios with a lower complexity.

#### 4.2.2. Outdoor Relocalization

We also conducted equivalent tests on the wayspots outdoor dataset, which is derived from the MapFree dataset [35] and comprises ten consecutive outdoor scenes. Following the testing methodology mentioned in the ACE paper, we utilized scenes 200–209 from the MapFree dataset, with each scene containing 580 training images and 580 testing images. The experimental results are displayed in Table 5 and Table 6. Although both our model and the original model struggled to achieve results comparable to those for the indoor datasets in outdoor settings, relatively speaking, our model’s camera localization performance proved superior to that of the original model in most parts of the dataset.

#### 4.2.3. Scene Reconstruction Results

**Figure 4:**
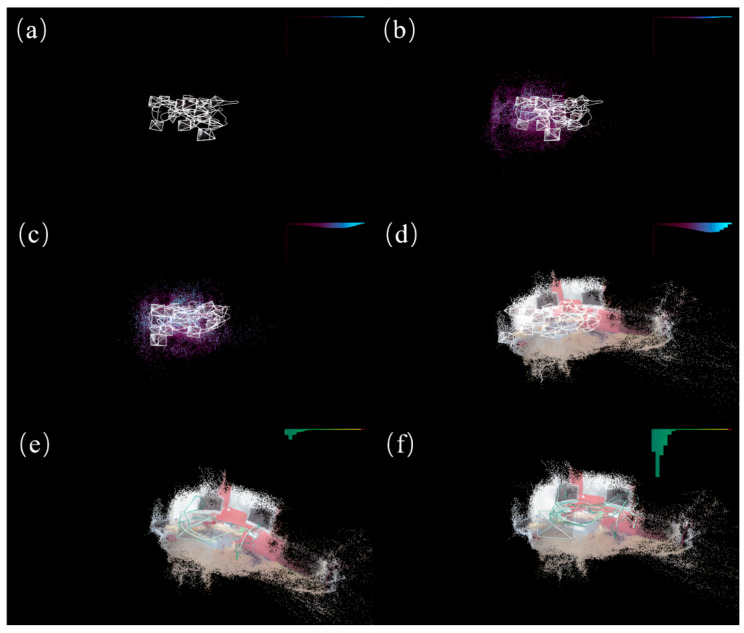
The scene reconstruction process and results for the chess scene of the 7scenes dataset using the improved model. (**a**) shows the scene reconstruction during the initial phase of model training, (**b**,**c**) depict the scene reconstruction during the model-training process, (**d**) illustrates the scene reconstruction after the completion of model training, and (**e**,**f**) display the camera localization and scene reconstruction during model testing, with the bar graph in the top-right corner representing the percentage of pixel offsets within various segmented thresholds.

We reconstructed the chess scene from the 7scenes dataset using our improved model. Given that scene reconstruction requires depth information predicted from all the pixels, we provisionally employed the previous loss function for this task. As the primary objective of the model is to enable faster and more accurate camera relocalization, which does not necessitate scene reconstruction, we retained the improvements made to the loss component within the model. In Figure 4, we present the process and results of scene reconstruction using the improved model on the chess scene from the 7scenes dataset.

### 4.3. Ablation Studies

Subsequently, we conducted isolated ablation studies on the two innovative aspects of our model using the 7scenes dataset, to separately validate their effectiveness and analyze the underlying reasons.

#### 4.3.1. Head and Loss Function Improvements

In this section, we discuss improvements made to the model’s head and loss function while retaining the original pose solver. Table 7 shows the experimental results of the model on the 7scenes dataset. It is evident that the model achieved an improved accuracy in camera localization. Unlike the original model, which utilized three serial skip connections in its head, our improved version included parallel skip and dense connections. Skip connections help mitigate performance degradation and address potential issues with vanishing or exploding gradients, while dense connections exploit the network’s feature information to enhance gradient flow. The parallel structure allowed both to exert their respective advantages, improving the overall representational power and optimization efficiency, thereby enhancing the model’s performance. Furthermore, we incorporated the calculation of Euclidean distance into the loss function for pixels outside a certain threshold, in addition to the existing Manhattan distance. The amalgamation of these two distance measures allowed the loss function to achieve a more comprehensive assessment of distances, adapting to various data distributions.

However, as one can see from Table 7, the improved head required additional time for some scenes only. One reason for this is the increased number of parameters; we calculated the parameters for the head component and found that, while the original model had approximately 2.1 million parameters in its head, the improved model’s parameter count exceeded 2.2 million. On the other hand, compared to residual connections, dense connections require more computational resources, and to enhance the performance further, we integrated a channel attention mechanism into the head, which also added to the time required. Consequently, to compensate for this increased time expenditure and achieve faster computational results, we improved the loss function by restructuring the program to delay pixel offset computations, thereby reducing the overall computational load. Additionally, we enhanced the pose solver, resulting in an accelerated resolution process.

#### 4.3.2. Pose Solver Enhancements

In this section, we report on the enhancements made to the pose solver part of our model, while retaining the original head. Table 8 shows the experimental results for the 7scenes dataset. It is observable that there was an increase in the computational speed during the camera localization by the model. Compared to the DSAC*-based pose solver used in the original model, our improved solver integrated two pose-estimation methods: SQPnP and ITERATIVE. SQPnP can solve for camera poses more quickly and directly via sparse linear systems, but is slightly less accurate than ITERATIVE, which solves for camera poses with a greater precision through iterative optimization, albeit at a cost to real-time performance. The pose-estimation method utilized by this model sequentially invoked both techniques, comparing the pre- and post-optimization offsets with a threshold of 0.01. SQPnP was selected for substantial corrections to the camera pose, while ITERATIVE was employed when incremental adjustments neared the accurate value. Additionally, we imposed further constraints on the early termination of the pose-estimation process. The previous approach terminated early only if the pixel displacement remained below a threshold. We introduced a criterion where, if the mean pixel displacement prior to iteration exceeded 1.2 times that following iteration, the process terminated early as well, effectively addressing issues where pursuing the accuracy of discrete points results in overall larger deviations, thus achieving more precise and faster localization in certain scenarios.

As indicated by Table 8, the pose solver that integrated ITERATIVE and SQPnP effectively enhanced the speed of pose estimation. It compensated for the computational slowdown caused by the improved head component without noticeably affecting the accuracy of pose estimation.

## 5. Conclusions

In this paper, we proposed a novel network model that rapidly relocalizes cameras in three dimensions using RGB images. This model improves upon the ACE model with refinements to the head section, the loss computation, and the pose-estimation components, achieving higher camera localization precision and a faster localization speed compared to the original ACE model. Firstly, for the head section, we restructured it by integrating dense connections, skip connections, and channel attention mechanisms, replacing the previous two-layer skip connections. Subsequently, we rewrote the loss calculation code, adjusting the order of coordinate offset computations that map to the pixel coordinate system to reduce the computational load. Simultaneously, we incorporated both Euclidean and Manhattan distances as losses for significant coordinate offsets, enhancing the model’s generalizability. Lastly, we modified the pose solver by adopting the AP3P method instead of the conventional P3P within RANSAC pose-estimation hypotheses. In the subsequent iterative optimization process, we integrated SQPnP and ITERATIVE methods, replacing the previous ITERATIVE pose solution, and added early-exit conditions during pose iteration optimization to mitigate the risk of converging to local optima. Ultimately, this allowed us to achieve faster pose estimation while maintaining the accuracy, better suiting the needs of real-world applications. The model was validated on the 7scenes, 12scenes, and wayspots datasets, demonstrating that the improvements in our model exhibited a certain level of generalizability across different scenarios. Furthermore, the ablation experiments elucidated the theoretical basis for the improvements, further confirming the effectiveness of our enhancements. Our work still has limitations. On one hand, the model was only trained and tested on datasets and has not yet been evaluated in real-world application scenarios, which will be the focus of our future work. On the other hand, similar to most previous models, our model still struggles to achieve a high localization accuracy under significant outdoor lighting variations, and it cannot guarantee robust generalizability across diverse environments. In the future, we will further refine the model to enhance its performance under varying lighting conditions and improve its environmental adaptability.

## Figures and Tables

**Table 1:** Comparison of relocalization results for the 7scenes dataset between the improved model and previous camera relocalization methods.

	Chess	Fire	Heads	Office	Pumpkin	Redkitchen	Stairs
PoseNet [34]	4.5°/0.13 m	11.3°/0.27 m	13.0°/0.17 m	5.6°/0.19 m	4.8°/0.26 m	5.4°/0.23 m	12.4°/0.35 m
SANet	0.9°/0.03 m	1.1°/0.03 m	1.5°/0.02 m	1.0°/0.03 m	1.3°/0.05 m	1.4°/0.04 m	4.6°/0.16 m
KFNet	0.7°/0.02 m	0.9°/0.02 m	0.8°/0.01 m	0.7°/0.03 m	1.0°/0.04 m	1.2°/0.04 m	1.0°/0.03 m
DSAC++	0.5°/0.02 m	0.9°/0.02 m	0.8°/0.01 m	0.7°/0.03 m	1.1°/0.04 m	1.1°/0.04 m	2.6°/0.09 m
DSAC*	0.7°/0.02 m	1.0°/0.03 m	1.3°/0.02 m	1.0°/0.03 m	1.3°/0.05 m	1.5°/0.05 m	49.4°/1.9 m
Ours	0.7°/0.02 m	0.8°/0.02 m	0.6°/0.01 m	0.8°/0.03 m	1.1°/0.04 m	1.3°/0.04 m	1.1°/0.04 m

**Table 2:** Test results for the 7scenes dataset using the improved model compared to the original model.

Error Range	Chess	Fire	Heads	Office	Pumpkin	Redkitchen	Stairs
10 cm/5 deg (%)	100/100	99.7/99.8	100/100	98.0/97.8	88.8/87.4	91.9/91.3	93.8/93.1
5 cm/5 deg (%)	96.7/96.6	92.5/93.3	99.7/99.4	86.6/85.0	59.2/58.2	61.2/59.3	68.6/70.2
2 cm/2 deg (%)	55.4/52.3	56.0/54.6	92.4/90.3	30.2/28.8	15.0/15.2	14.1/13.3	9.8/8.3
1 cm/1 deg (%)	18.1/16.9	16.1/13.8	54.0/49.6	6.5/6.0	3.5/3.1	2.6/1.8	1.3/1.2
Rotation (deg)	0.7/0.7	0.8/0.9	0.6/0.7	0.8/0.8	1.1/1.1	1.3/1.4	1.1/1.1
Translation (cm)	1.8/1.9	1.8/1.9	0.9/1.0	2.7/2.8	4.3/4.4	4.2/4.3	3.9/3.8
Avg. time (ms)	30.5/34.6	34.4/37.8	32.6/35.9	33.6/38.6	36.8/40.8	38.5/41.9	41.3/42.6

**Table 3:** Test results for the first six scenes in the 12scenes dataset, comparing the improved model with the original model (ours/ACE).

Error Range	apt1_kitchen	apt1_living	apt2_bed	apt2_kitchen	apt2_living	apt2_luke
10 cm/5 deg (%)	100/100	100/100	100.0/100	100/100	100/100	100/100
5 cm/5 deg (%)	100/100	100/100	100.0/100	100/100	100/100	99.4/99.0
2 cm/2 deg (%)	99.2/98.3	84.8/83.0	91.2/89.2	97.1/96.2	92.8/92.3	78/76.3
1 cm/1 deg (%)	68.3/70.3	47.7/46.7	57.8/52.5	72.4/66.2	62.2/63.6	34.5/31.9
Rotation (deg)	0.4/0.4	0.4/0.4	0.4/0.4	0.4/0.4	0.3/0.3	0.6/0.6
Translation (cm)	0.7/0.7	1.0/1.1	0.9/1.0	0.8/0.8	0.8/0.8	1.3/1.3
Avg. time (ms)	22.4/36.1	21.4/34.4	22.5/38.2	19.9/32.7	21.4/32.6	24.5/38.2

**Table 4:** Test results for the last six scenes in the 12scene dataset, comparing the improved model with the original model (ours/ACE).

Error Range	Office1-gates362	Office1-gates381	Office1-Lounge	Office1-Manolis	Office2-5a	Office2-5b
10 cm/5 deg (%)	100/100	100/100	100/100	100/100	100/100	100/100
5 cm/5 deg (%)	100/100	99.9/99.1	100/100	100/100	98.2/97.0	99.5/100
2 cm/2 deg (%)	92.0/90.7	86.0/83.4	85.9/83.8	92.6/90.8	81.7/79.5	76.3/74.8
1 cm/1 deg (%)	54.7/54.9	38.7/37.2	35.2/33.9	56.0/56.7	35.6/38.6	36.0/35.1
Rotation (deg)	0.4/0.4	0.5/0.6	0.4/0.4	0.4/0.4	0.5/0.5	0.4/0.4
Translation (cm)	0.9/0.9	1.2/1.2	1.3/1.3	0.9/0.9	1.2/1.2	1.3/1.3
Avg. time (ms)	22.6/37.4	27.8/40.3	21.8/34.4	23.1/34.3	25.3/37.7	23.7/35.2

**Table 5:** Test results for the first five scenes in the wayspots dataset, comparing the improved model with the original model (ours/ACE).

Error Range	Bears	Cubes	Inscription	Lawn	Map
10 cm/5 deg (%)	80.0/79.1	87.0/89.7	51.2/42.5	35.1/35.4	56.1/56.1
5 cm/5 deg (%)	67.1/66.2	30.1/41.9	20.7/17.3	23.5/23.7	37.2/34.0
2 cm/2 deg (%)	9.0/6.2	3.8/5.9	1.8/1.8	2.1/0.3	3.4/3.0
1 cm/1 deg (%)	0.5/0.7	0.0/1.0	0.0/0.0	0.0/0.0	0.0/0.0
Rotation (deg)	1.1/1.2	0.8/0.8	1.5/1.6	37.0/30.9	1.2/1.2
Translation (cm)	3.6/3.8	6.5/5.7	9.7/12.0	124.3/128.7	7.0/7.6
Avg. time (ms)	85.5/84.9	40.8/46.4	78.3/81.2	108.3/109.3	46.1/47.3

**Table 6:** Test results for the last five scenes in the wayspots dataset, comparing the improved model with the original model (ours/ACE).

Error Range	Squarebench	Statue	Tendrils	Therock	Wintersign
10 cm/5 deg (%)	66.6/64.0	0.0/0.0	34.6/33.0	98.4/100	0.9/0.7
5 cm/5 deg (%)	43.0/42.6	0.0/0.0	5.3/5.7	63.7/75.6	0.0/0.0
2 cm/2 deg (%)	6.1/6.4	0.0/0.0	0.0/0.0	19.6/30.1	0.0/0.0
1 cm/1 deg (%)	0.3/0.0	0.0/0.0	0.0/0.0	6.7/7.4	0.0/0.0
Rotation (deg)	0.7/0.7	13.1/15.6	45.2/49.9	0.8/0.8	0.9/1.1
Translation (cm)	5.9/6.0	476.9/576.2	165.8/183.5	3.9/3.1	388.2/484.7
Avg. time (ms)	41.0/42.6	120.0/126.9	104.9/104.0	21.5/29.7	126.7/123.7

**Table 7:** Performance results for the 7scenes dataset using the model with improvements to only the head section and loss function compared to the original model (our_head/ACE).

Error Range	Chess	Fire	Heads	Office	Pumpkin	Redkitchen	Stairs
10 cm/5 deg (%)	100/100	99.8/99.8	100/100	98.0/97.8	88.5/87.4	91.7/91.3	93.4/93.1
5 cm/5 deg (%)	96.8/96.6	93.2/93.3	99.9/99.4	86.2/85.0	59.2/58.2	59.7/59.3	71.8/70.2
2 cm/2 deg (%)	54.3/52.3	56.1/54.6	91.4/90.3	29.1/28.8	16.3/15.2	13.6/13.3	10.1/8.3
1 cm/1 deg (%)	15.7/16.9	16.0/13.8	51.1/49.6	7.1/6.0	3.4/3.1	2.2/1.8	1.3/1.2
Rotation (deg)	0.7/0.7	0.8/0.9	0.6/0.7	0.8/0.8	1.1/1.1	1.3/1.4	1.1/1.1
Translation (cm)	1.9/1.9	1.8/1.9	1.0/1.0	2.7/2.8	4.2/4.4	4.3/4.3	3.7/3.8
Avg. time (ms)	35.6/34.6	38.2/37.8	34.1/35.9	37.4/38.6	40.6/40.8	42.1/41.9	41.8/42.6

**Table 8:** Performance results for the 7scenes dataset using the model with improvements to only the pose solver compared to the original model (our_PnP/ACE).

Error Range	Chess	Fire	Heads	Office	Pumpkin	Redkitchen	Stairs
10 cm/5 deg (%)	100/100	99.8/99.8	100/100	97.8/97.8	88.2/87.4	91.9/91.3	93.7/93.1
5 cm/5 deg (%)	96.9/96.6	93.2/93.3	99.6/99.4	85.5/85.0	57.6/58.2	59.3/59.3	67.6/70.2
2 cm/2 deg (%)	52.2/52.3	53.2/54.6	90.6/90.3	29.5/28.8	14.6/15.2	13.1/13.3	9.5/8.3
1 cm/1 deg (%)	17.4/16.9	16.4/13.8	49.6/49.6	6.1/6.0	3.7/3.1	2.0/1.8	1.3/1.2
Rotation (deg)	0.7/0.7	0.8/0.9	0.6/0.7	0.8/0.8	1.1/1.1	1.3/1.4	1.2/1.1
Translation (cm)	1.9/1.9	1.9/1.9	1.0/1.0	2.8/2.8	4.4/4.4	4.4/4.3	4.1/3.8
Avg. time (ms)	30.3/34.6	32.6/37.8	32.3/35.9	33.3/38.6	35.7/40.8	37.0/41.9	37.8/42.6

## Data Availability

The data are contained within the article.
